# Uncharacteristic metastasis of a renal clear-cell carcinoma to the muscles of the forearm—case report

**DOI:** 10.1259/bjrcr.20150495

**Published:** 2017-03-10

**Authors:** Oliwia Kozak, Paweł Turzyński, Karolina Markiet, Joanna Pieńkowska, Katarzyna Skrobisz-Balandowska, Michał Studniarek

**Affiliations:** ^1^Department of Radiology, Medical University of Gdansk, Gdansk, Poland; ^2^2nd Department of Radiology, Medical University of Gdansk, Gdansk, Poland

## Abstract

Clear-cell renal carcinoma constitutes over 90% of all cases of renal cancers. One of the least common locations of metastases of this type of cancer are skeletal muscles. We believe that this is the first case ever presented of renal clear-cell cancer metastasis to the extensor digitorum muscle. Our case should sensitize clinicians and radiologists dealing with this type of carcinoma to the abundance of metastases, their uncommon locations and frequently asymptomatic nature, which all makes them easy to overlook on physical and imaging examinations.

## Summary

Remote metastases and their atypical locations are characteristic for clear-cell renal carcinoma. They can be observed even after a very long time from removal of the primary lesion. Metastases to skeletal muscles are very rare and a single metastasis to a finger extensor is, according to the authors’ best knowledge and belief, the first case ever reported. Our patient presented with three uncommon metastases of clear-cell carcinoma—to the pancreas, thyroid and a finger extensor, which supports the unpredictable biology of this type of carcinoma.

A 67-year-old male patient with a history of clear-cell renal carcinoma was admitted to the Department of Orthopaedics of the University Clinical Centre with suspected metastasis in the right forearm. MRI showed metastasis to the muscles of the forearm, while a core-needle biopsy confirmed the diagnosis of clear-cell renal carcinoma. The lesion was excised during a non-complicated surgery.

## Clinical presentation

We present a case of a 67-year-old male patient admitted to our tertiary hospital because of a 6-month history of painful right forearm secondary to suspected bony metastasis. The patient’s medical history included arterial hypertension, ischaemic heart disease, Type 2 diabetes and psoriasis. In 2011, he underwent radical nephrectomy owing to clear-cell renal carcinoma. In 2013, resection of the head of the pancreas was conducted owing to neoplastic metastasis. PET examination carried out in 2014 revealed clear-cell carcinoma metastasis to the right lobe of the thyroid and strumectomy was performed. Another physical examination revealed a palpable tumour of 6 × 4 cm in size on the right forearm.

## Differential diagnosis

Clear-cell renal carcinoma frequently metastasizes to bones. A tender palpable tumour on the forearm is consistent with a lesion of a metastatic type.

Differential diagnosis should include other neoplasms of soft tissues, which are probable to occur at this age such as chondrosarcomas, lipomas, liposarcomas or fibrosarcomas.

## Investigation/imaging findings

**Figure 1.**
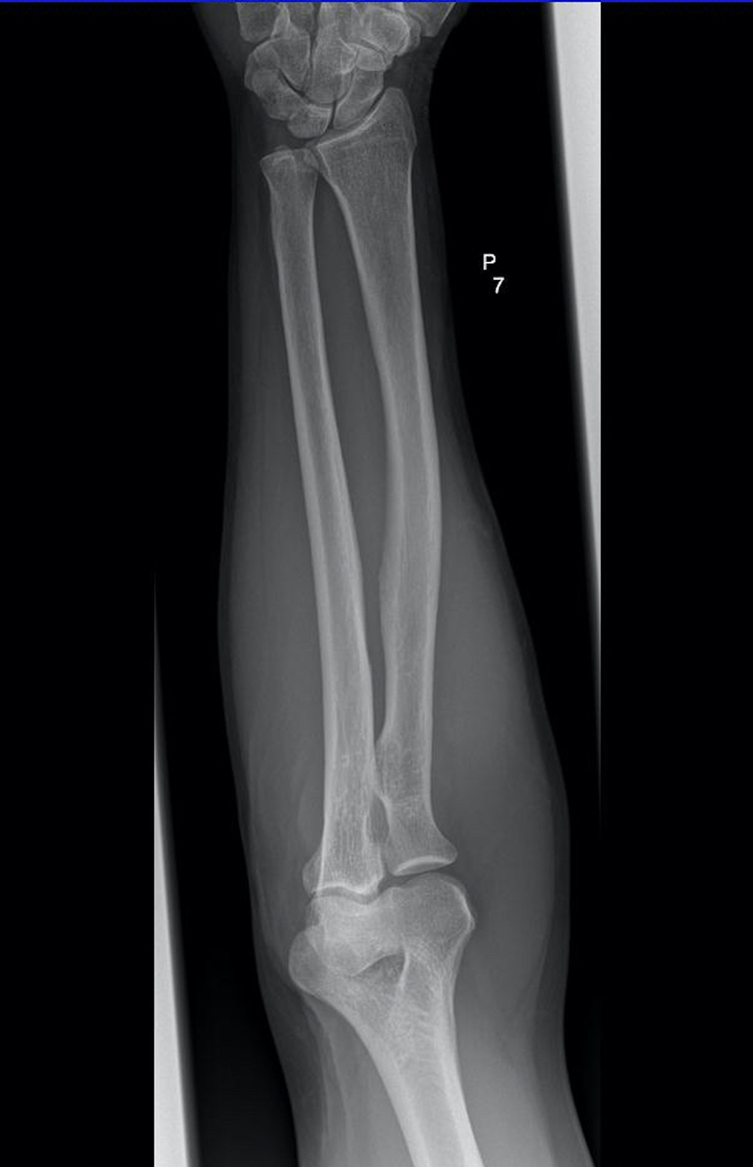
Plain AP radiograph of the forearm and elbow showed no obvious soft tissue mass in the region of extensor digitorum muscle or evidence of any bone destruction.

An X-ray scan of the forearm revealed no significant anomaly in the projection of the coronoid process of the ulna (Figure 1).

**Figure 2.**
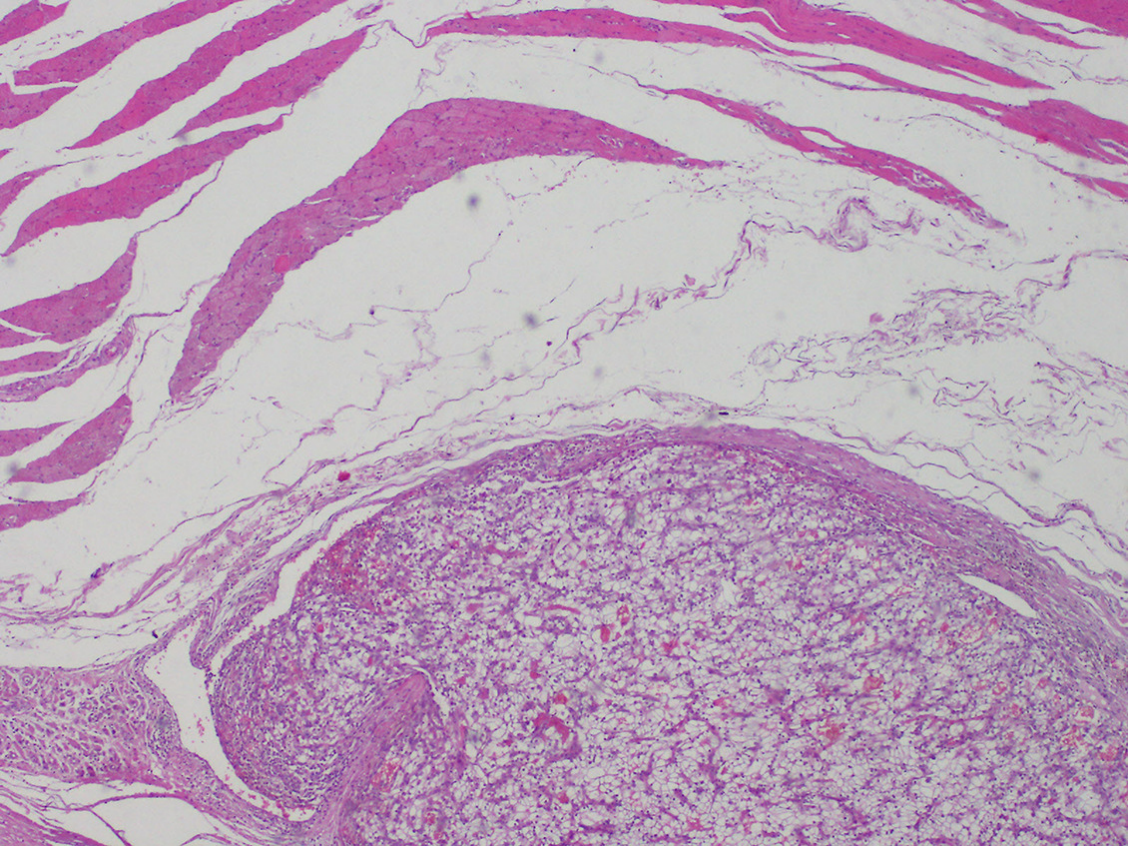
Histopathological examination of the excised specimen showed typical cells for RCC in the muscle tissue.

**Figure 3.**
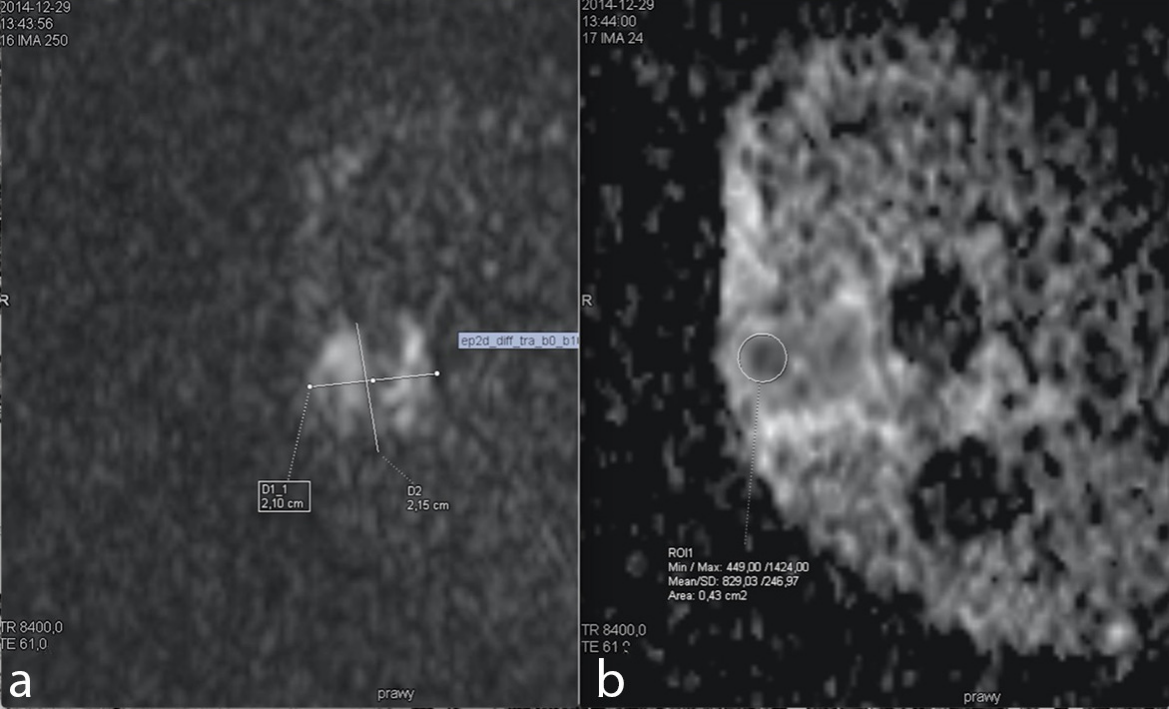
Diffusion Weighted Imaging revealed significant restriction within the pathological mass (a). Apparent Diffusion Coefficient (ADC) measurement recorded for a marked region of interest (ROI) (b).

Further evaluation by MRI revealed a heterogeneous focal infiltration measuring 66 × 24 × 19  mm in size located in the extensor digitorum muscle of the right forearm with no additional lesions or involvement of the adjacent bones. The infiltration extended beyond the fascia of the muscle and penetrated 10 mm between the extensor carpi ulnaris and supinator. After administration of contrast medium the focus was significantly non-homogenously enhanced, which is typical for secondary lesions of renal cell carcinoma (Figures 2 and 3). Core-needle biopsy confirmed clear-cell renal carcinoma metastasis.

## Treatment, outcome and follow up

**Figure 4.**
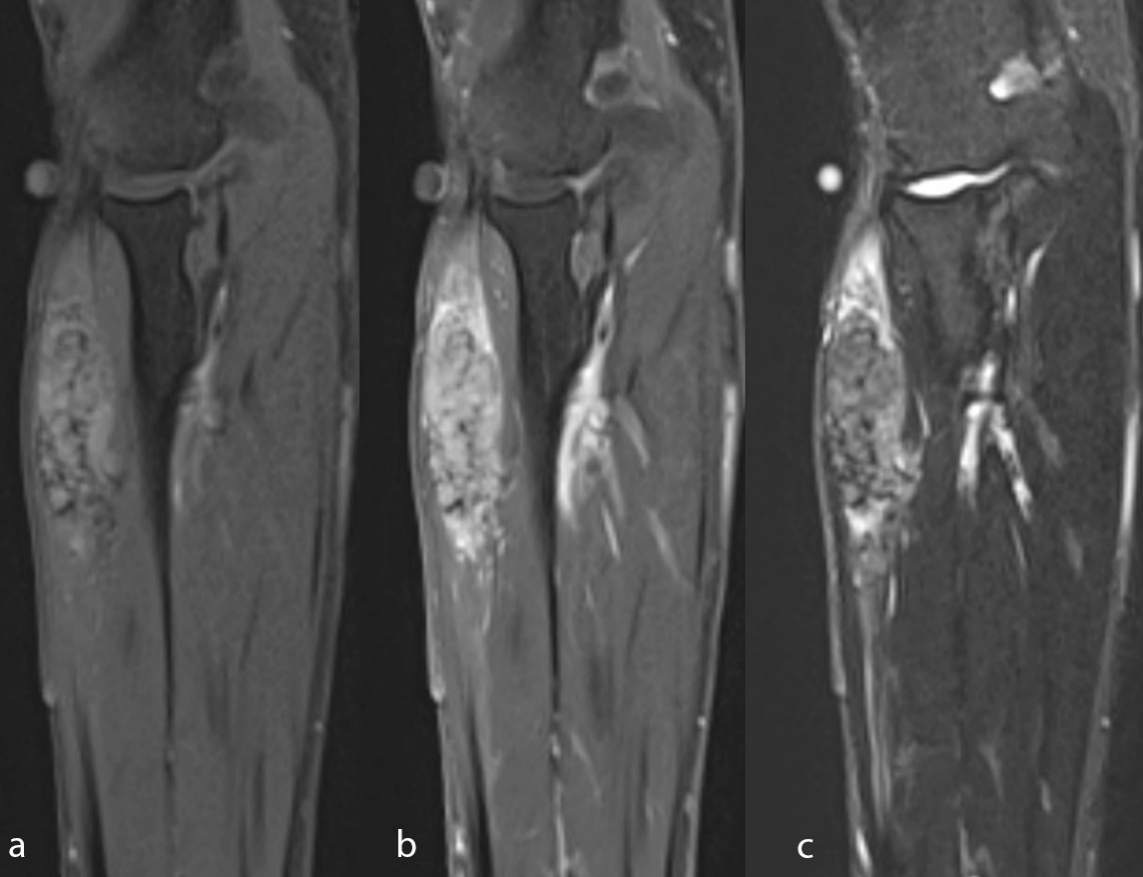
MRI coronal images present pathological mass in the extensor digitorum muscle corresponding to CCRCC metastasis in *T*_1_ weighted fat-saturated pre-contrast image (a) *T*_1_ weighted fat-saturated post-gadolinium image (b) and *T*_2_ weighted fat-saturated image (c) respectively.

The lesion was excised and the perioperative course was non-complicated. The metastatic nature of the lesion was confirmed after histopathological examination of the excised specimen (Figure 4). Eight months after the surgery a follow-up ultrasound examination of the operated site showed no pathologies.

## Discussion

The prevalence of malignant neoplasms of the kidney varies geographically, with the highest incidence in Europe and North America which ranges 2.9–15.2/100 000 in males and generally half of those cases in females. Risk factors for renal carcinoma include nicotinism, arterial hypertension, obesity, overuse of analgesics, polycystic kidneys and occupational exposure to toxic substances such as cadmium and asbestos.^1^ Clear-cell renal carcinoma can also be of genetic aetiology in the case of which it can occur in the course of von Hippel–Lindau disease with neoplasms located in the cerebellum, retina and adrenal medulla.

Owing to high vascularization, renal carcinomas show great ability to metastasize, mainly along the blood circulation system. Most prevalent are metastases to lungs (50%), lymph nodes (35%), liver (30%), bones (30%), adrenal glands (5.5%) and brain (2%).^2^ What is characteristic of this type of carcinomas are the uncommon locations of metastases such as salivary glands, heart, oesophagus or hard palate. Metastases to skeletal muscles are extremely rare. This is because of the high vascularization of the muscles, their extensive surface and production of lactic acid which suppresses tumour’s angiogenesis.^3^ Metastases to muscles are most frequently observed in the course of lung cancer.^4^ The prevalence of metastases of renal carcinoma to skeletal muscles is estimated at 0.4%.^5^ Their actual prevalence is probably much higher; however, it is difficult to assess owing to the fact that metastases to skeletal muscles can remain asymptomatic for a long time. Owing to their low prevalence they may be overlooked during preparation of radiological impression, even by highly experienced radiologists. The metastases are usually detected only when they reach a large size and start to exhibit symptoms.

In literature, cases of metastases of the renal cell carcinoma to the following muscles have been described: deltoid, triceps brachii, biceps, brachioradialis, muscles of the scapula, trapezius, muscles of the abdominal wall, iliacus, iliofemoral muscle, gluteus maximus, gluteus medius, quadriceps femoris, biceps femoris, adductor magnus and sartorius.

Metastases of the renal cell carcinoma to the muscles are characterized by clearly increased signal intensity in *T*_2_ weighted MRI images, a similar enhancement as primary tumor which can be homogenous, heterogeneous or peripheral and in USG they mostly appear as well-lobulated, heterogeneous, hypoechoic referred to muscles changes.

Untreated patients with metastatic RCC have a median survival of 6 to 12 months and a 5 year survival rate of <20%.^6^ RCC muscle metastases are known only from case reviews, so there is no particular study which compares the treatments results or provides any treatment recommendations; however the survival rate in atypical metastases is comparable with lung metastases.^7^ Early detection of single metastases is crucial since it allows for application of surgical treatment. Excision of metastatic lesions improves the prognosis, increasing the 5 year survival rate by approximately 35–50%.^8^ A survival advantage from complete metastasectomy was also observed among patients with multiple, non-lung-only metastases, who had a 5 year survival rate of 32.5% with complete resection versus 12.4% without complete resection.^9^ There is also a potential benefit from integration of metastasectomy and immunotherapy, with the median survival rate of 4.7 years and 1.8 years of median time to progression.^10^ In patients treated with systemic combinational targeted and immunotherapy using tyrosine kinase inhibitors, interferon-α, interleukin 2 and 5FU or vinblastine, the mean progression-free survival was 8.1 months with overall survival 16.8 months.^11^ Because of the unpredictable character of RCC, it is impossible to provide for an accurate follow up in this group of patients, but careful clinical examination and control abdomen CT should be considered as most effective. CT including nephrographic phase is the most accurate method for observation of detected metastases and for monitoring of response to treatment. Patients with renal carcinoma should be observed for many years, since this type of cancer can metastasize even after 19 years from the moment of excision of the primary tumour.^12^

## Learning points

1. Clinicians and radiologists dealing with patients who suffer from clear-cell renal carcinoma should always bear in mind the unpredictable nature of this neoplasm and its high ability to metastasize to uncharacteristic locations such as skeletal muscles
2. The metastases are frequently asymptomatic.
3. Early detection of metastases allows for surgical treatment and thus improves the prognosis.

## Consent

The informed consent was obtained to publish this case. 
